# What makes an effective grants peer reviewer? An exploratory study of the necessary skills

**DOI:** 10.1371/journal.pone.0232327

**Published:** 2020-05-13

**Authors:** Miriam L. E. Steiner Davis, Tiffani R. Conner, Kate Miller-Bains, Leslie Shapard

**Affiliations:** 1 Research Services, Research, Reviews, Evaluation, and Technology, Oak Ridge Associated Universities, Oak Ridge, Tennessee, United States of America; 2 Assessment & Evaluation, Research, Reviews, Evaluation, and Technology, Oak Ridge Associated Universities, Oak Ridge, Tennessee, United States of America; University of Toronto, CANADA

## Abstract

This exploratory mixed methods study describes skills required to be an effective peer reviewer as a member of review panels conducted for federal agencies that fund research, and examines how reviewer experience and the use of technology within such panels impacts reviewer skill development. Two specific review panel formats are considered: in-person face-to-face and virtual video conference. Data were collected through interviews with seven program officers and five expert peer review panelists, and surveys from 51 respondents. Results include the skills reviewers’ consider necessary for effective review panel participation, their assessment of the relative importance of these skills, how they are learned, and how review format affects skill development and improvement. Results are discussed relative to the peer review literature and with consideration of the importance of professional skills needed by successful scientists and peer reviewers.

## Introduction

Science relies on accurate, efficient, and measured peer review of research. Peer review is the de facto standard in decision-making for most funding bodies [1], and is the gold standard [2,3] for evaluating scientific merit in decision-making regarding research funding [4]. Roberts and Shambrook [5] describe peer review as “essential to academic quality, fair and equitable, and one of the most rigorous and prestigious forms of scholarly accomplishment.” Guthrie, Ghiga, and Wooding [6] found “good evidence” that peer review has the support of scientific stakeholders. However, work subjected to peer review may or may not be of greater quality than work not subjected to peer review [5], and is a weak predictor of future success [6]. One possible reason for the variability in scores and prediction is that the peer review process relies on people, people are fallible [7] and “subject to the influence of personalities” [8]. In fact, according to Towne, Fletcher, and Wise [9] “the peer review process, no matter how well designed, is only as effective as the people involved.” With respect to panel reviews, Towne et al. [9] state, “assembling the group of reviewers is the very crux of the matter” [9]. Additional criticisms of grants and funding peer review stem from perceptions of bias against innovative research (conservatism), cronyism, failure to detect misconduct and malpractice, subjectivity, lack of accountability, inconsistency, incompleteness, and negativity towards interdisciplinary research [3,6,7]. While the Research Information Network [7] noted such criticisms are often directed at deficiencies in practice rather than in principle and concluded no better alternative means of research evaluation exist, Avin [3] summarized the criticisms of several scholars based not only on the practice of peer review but on the principle(s) as well. Countering the criticisms of conservatism in particular, Avin [3] and Guthrie et al. [6] suggest random allocation of at least a portion of research funding rather than by means of peer review.

Despite varied opinions on the merits of peer review as a means of advancing science, the ubiquitous practice of peer review, as a method of deciding upon and awarding research funding, remains relatively understudied [3,6,10]. Kostoff [11] notes the need for studies on the relationships of cost to quality, evaluations to research improvement, training to quality, training to reliability, and training to validity. Carpenter, Sullivan, Deshmukh, Glisson and Gallo [1] suggest the need for research on decision-making, teamwork, and the effect of review format on scoring and discussion of grant applications. Guthrie et al. [6] suggest research on the social processes of peer review and panel meetings. And Gallo et al. [10] remarked that there is a great deal of subjectivity in the evaluation of research applications; understanding the sources of, and relative contributions to, reviewer disagreement is crucial to improve the peer review process.

The bulk of literature available concerning peer review conducted for research funding agencies predominantly focuses on outcomes in terms of funding decisions and whether peer reviewed research produces better science than non-peer reviewed research. Coveney, Herbert, Hill, Mow, Graves, and Barnett [12] considered the definitions of “good science” among panel reviewers in different fields. Some authors have pointed to the importance of the review process [12] and the people serving as panelists [13],—either the need to identify and understand them nor their significance to the success of peer review panels. When panel format has been examined, it has been from the perspective of determining if using technology produces better outcomes [14,15] in decision-making [16] or merit scores, score distribution, or reviewer demographics [17,18]. Answers have been inconclusive and resulted in numerous calls for further research on the effects of panel format, yet these suggestions of additional investigation rarely reference peer review skills.

Also missing from the literature is information about what improves review panelists’ skills, and whether different review formats have different effects on skill development or improvement. Development of reviewers’ skills, and in turn their effect on panel review outcomes and decision-making, is rarely mentioned in the literature. Mow [19] describes the characteristics panel members use to assess proposals, suggesting elements of panel review elements that funding agencies may benefit from, including characteristics on which reviewer skill development could be supported as they seek to improve the research funding review process and evaluate outcomes. Coveney et al. [12] and Turner, Bull, Chinnery, Hinks, Mcardle, Moran, Payne, Woodford Guegan, Worswick, and Wyatt [20], conducted qualitative studies capturing first person reports from peer review panelists concerning the peer review process, fairness, and the criteria used in decision-making. While skills can be extrapolated from Coveney et al. [12] (group dynamics), Mow [13] (definitions of excellence, interaction), Turner et al. [20] (time, good reviewer, value), and Bol [21] (writing, using tools); only Porter [22] (skimming, big picture, discernment), Member [23] (how to prepare, one’s role, utilizing program guidelines), and Irwin, Gallo, and Glisson [24] (efficiency, writing, decision-making, evaluation) explicitly discuss reviewers’ perspectives on panelist skill(s). Thus, this exploratory study sought to add additional perspectives to the literature and focused on three questions:

1. What skills are important for peer review panelists reviewing proposals for research funding?
2. What is the effect on review panelist skill development and improvement in two formats, face-to-face and virtual reviews?
3. What other activities develop or improve panel reviewer skills?

Synchronous and interactive panel peer reviews were considered in two formats: virtual (all panelists participate at the same time, but not in the same place) and face-to-face (all panelists participate at the same time and in the same place). Although the researchers recognize that blended panels (online and face-to-face concurrently) are becoming more common, they were purposely excluded in this exploration.

## Literature review

The review of literature focuses on two specific areas: panelist skills and panel format (face-to-face and virtual). Assuming that what is taught, or the criteria that is used, indicates what is believed necessary for effective participation and success, the types and content of training offered to reviewers are also considered.

### Panelist skills

While there is not a robust body of literature on peer review skills, there are pertinent studies by Markin [8], Towne et al. [9], Coveney et al. [12], Congressionally Directed Medical Research Program (CDMRP) [25], and Hackett and Chubin [26] on the peer review process and by Rivard, O’Connell and Wegman [27] and the Peer Review Task Force [28] on agency best practice guidelines. These studies and guides revealed several desired, and implied, reviewer skills and characteristics. This section provides detail on skills highlighted in the literature.

The most commonly referenced skills are subject matter expertise, and excellence and achievement (track record) as indicated by publications, funding records, awards, positions and/or patents. Panels are typically made up of “scientific experts,” [25] those with “expertise in the field of activity relevant to the proposals,” [8] and excellence and achievement in their fields [27]. Fogelholm, Leppinen, Auvinen, Raitanen, Nuutinen and Väänänen [29] maintain that peer review relies upon reviewer expertise to evaluate quality, validity, relevance, and potential for innovation. With reference to in-person peer review panels of grant proposals, those interviewed by Coveney et al. [12] not only reference the need for panels to contain “significant expertise” but also that “the panel must be selected to ensure a broad range of experiences.”

Among the additional skills identified as necessary for effective grants’ peer review are communication, time management, interpersonal skills, writing, critical thinking, problem solving, and decision-making. Gallo et al. [10] state that writing, critical thinking, and speaking skills were necessary. Woods, Briedis, and Perna [30] referenced communication skills, critical thinking, and problem solving. Effective participation in a panel review also requires subject matter expertise [7,9,10], proficiency in writing, critical thinking, and speaking [31], independent thinking [8], preparation, humility, fairness, willingness to change, and discretion [17]. Irwin et al. [24] and Porter [22] found specific skills develop from, or are improved by, participation in a panel review including efficiency, discernment, evaluation, knowledge in grant writing, and decision-making ability.

In one of the first person reviewer narratives, Member [23] emphasizes the importance not only of preparation, but also of knowing how to prepare. Turner et al. [20] focused on process and training to be a “good reviewer”. Skills extrapolated from studies on process improvements are time management and expectations of time commitment [32], how to focus on strengths, weaknesses, and flaws [20], understanding conflict of interest [20,33], and measuring expertise [19].

The Peer Review Task Force in the Office of Energy Efficiency and Renewable Energy [28] requires reviewers to be “independent, competent, and objective” and to have “no real or perceived conflicts of interest.” Towne et al.’s [9] report describes several skills employed by effective reviewers including respectful listening, open mindedness, and a balance between dominance and acquiescence during discussions. Robert Sternberg [9] notes that “creativity is an undervalued yet critical talent for assessing research quality.” Coveney et al. [12] expressed the need to identify and be familiar with the research culture and to define excellence, while Turner et al. [20] discuss the need to define value.

Several authors have suggested skills that good reviewers should possess. Member [23] mentioned understanding one’s role as a panelist and panel functionality. Woods et al. [30] included verbal and written communication skills, critical thinking, and problem solving. Cheetham and Chivers [34] and Yen, Horner-Devine, Margherio, and Mizumori [35] mentioned networking and teamwork including the ability to collaborate and work well within diverse teams. Gallo et al. [10] referenced writing skills and critical thinking skills, and Vo et al. [17] mentioned fairness in grading and willingness to change scores based on the conversation. Woods et al. [30], Metcalfe, Thompson and Green [36], and Galland, McCutcheon, and Chronister [37] highlighted professional skills that scientists, and by connection reviewers, should possess were problem solving, ethics, collaboration, professionalism, self-discipline, self-efficacy, and innovation and integrity. In 2002, Metcalfe et al. [36] indicated the United Kingdom funding councils had produced the “most comprehensive generic list” of reviewer skills. These skills were categorized into research skills and techniques, research environment, research management, personal effectiveness, communication skills, networking and teamworking, and career management.

### Training

Literature addressing the training of peer reviewers of grants is sparse and pertains predominantly to the practices of specific organizations or to generalized and standard practices rather than the peer review skills necessary for effective reviewer participation. Below we showcase different topics and types of training offered to panelist from a variety of agencies and funding bodies.

The American Heart Association [33] reviewers “undergo extensive online training” about how to review a grant and identify conflicts of interest. The training described in the National Research Council’s 2004 report edited by Towne et al. [9] included general principles and policies, purpose, applying review criteria, model reviews, describing strengths and weaknesses, and using review criteria in assessments. CDMRP [25] notes that all reviewers receive training via online modules and webinars, including required training for first-time reviewers. “The webinars include an overview of the history of the research program; award mechanisms, corresponding program announcements, and peer review criteria to be used; and the logistics of the peer review panel meeting” [25]. Sattler, McKnight, Naney and Mathis [38] included information on the importance of the review process, how scores relate to funding decisions, the meaning of each value on the rating scale, and instruction on assigning scores and understanding review criteria. The British Academy [39] report indicates peer review training includes attention to academic quality, professional ethics, intellectual property, and fair consideration of work by colleagues. The report [39] recommends, “formal training in peer review and its principles be incorporated into the training guidelines of the Research Councils and [higher education] institutions.”

Chubin [40,41] and Kruytbosch [42] describe peer review as a system of social interaction and social ideology. As a social system, social skills are imperative and by definition, reviewers would need to possess such skills to interact effectively in group decision-making [12,19] to elucidate a successful panel review. The majority of such social skills are referred to as “professional” skills emphasizing both personal and professional effectiveness. Professional skills are defined as the “interpersonal, human, people, or behavioral skills needed to apply technical skills and knowledge in the workplace” [43] and the “cluster of personal qualities, habits, attitudes, and social graces that make someone a good employee and a compatible coworker” [44]. Although these skills are identified as among those effective reviewers need to draw upon, their utility in review, and the need to train reviewers in them, are disconnected. Only Guilford’s [45] article concerning manuscript peer review notes these professional skills are needed by researchers for the purpose of participation in peer review. The stakeholders Turner et al. [20] interviewed claimed training is needed on “how to be a good reviewer.” Current assumptions appear to be that the skills necessary for success in peer review will not only be acquired “along the way,” [46] but will be effectively present when needed.

Professional skills also include identifying and avoiding unconscious bias. Coveney et al. [12], Mow [13], Turner et al. [20], and Abdoul, Perrey, Amiel, Tubach, Gottot, Durand-Zaleski, and Alberti [30] all discuss track record as one important criterion used in panel reviews. Track record is determined by the number of publications, research team, institution, and/or past funding of an applicant which can exacerbate what Merton [47] referred to as the Matthew effect. The Matthew effect [21,47] favors those who have an advantage (track record criteria) while discriminating against those who have not had the same advantages. This type of unconscious bias is another area of training indicated by recent publications [19,21].

### Peer review panels and panel format

Review format has been considered in relation to factors such as scores, review quality, reliability, efficiency, team performance, communication patterns, and reviewer participation. While the prevailing sense is that face-to-face panels are the “gold standard” [48] there is little conclusive evidence to support this. It is unclear whether differences exist in review quality across formats and which format is “better” [16,29], but Pier et al. [14,16] did note slightly greater efficiency of reviewers’ time in face-to-face meetings compared to virtual meetings. Venkatraman [15] found a greater amount of, and more valuable, reviewer participation in face-to-face settings compared to virtual settings. Carpenter et al. [1] noted small but statistically significant differences between settings (face-to-face vs. teleconference, no visual component) in terms of its effect on discussion and posit “teleconference reviewers possibly being slightly less engaged than those participating onsite.” Graves, Barnett, and Clarke [49] examined panel size and the percent of funded proposals. They found that reliability was increased with larger panels. This suggests that virtual settings may be more cost effective for funding agencies.

Increasing costs and improvements in technologies such as teleconference, videoconference, webinars, virtual meetings, etc. have made virtual formats “a desirable forum compared to traditional face-to-face settings” [1], and promising for reducing costs [48]. Online panels save money and allow a greater number and variety of reviewers, including international scientists, to participate. Within virtual panels the work of evaluation is accomplished, “applications are read, and decisions are taken efficiently” [15]. The Canadian Institutes of Health Research [50] shifted from in-person to online reviews in 2014 to, among other things, “gain cost-effective access to a broader base of expertise (including international experts).” Advances have made studying the impact of technology on reviews and the question of whether technology can match the perceived quality of traditional in-person reviews particularly relevant [51] and under assessed [48].

Venkatraman [15] found that participation and the ability to recruit high-level reviewers increases with technological options. Online reviews enable the inclusion of a greater variety of researchers. For example online reviews can accommodate scientists who cannot travel or are located internationally [50,52]. Virtual panels also allow younger scientists, or those who do not have a travel budget, to participate equally [21].

Other researchers, however, claim virtual panel reviews negatively impact debate, confidentiality, and engagement. Webster [52] writes, “when you are together in a room, you are much more committed to the process than when it’s online.” Networking and collaborations can be missed in virtual panels, confidentiality is challenged, technological difficulties exist, and major disagreements are harder to resolve [15]. Similarly, virtual discussions may remove productive debate, support biases towards established researchers, and disallow creative discussion and collective visioning for a field [50]. Technology-enabled panels also have a greater tendency to increase a task-oriented focus [10], conform to norms [53], and require more awareness [54]. According to Gallo et al. [10] face-to-face panel reviews offer a “mentoring effect” which they argue benefits reviewers’ education as researchers, improves their own ability to obtain research funding, offers the opportunity to share ideas and learn from others, and embraces the collective effort to move science forward. According to Venkatraman [15] face-to-face panel reviews have been criticized for being primitive and environmentally irresponsible, and for providing less social benefit than purported.

While face-to-face panels are the standard in judging grant funding decisions [25], it is unclear whether differences exist in review quality across formats and if so, which format is “better” [6,16,29]. Multiple authors examine the value of discussion and trust in peer review panels as well as how they are impacted by panel format and use of technology. With respect to discussion, Obrecht, Tibelius, and Alosio [55] conclude it added no value over pre-meeting evaluations, and Fogelholm et al. [29] infer it did not increase the reliability of evaluation. However, Martin, Kopstein, and Janice [56] found discussion has an important and practical impact on peer review evaluations.

With respect to the impact of review format on discussion, Carpenter et al. [1] note a small but statistically significant difference in discussion effects based on review format, with a greater magnitude of the discussion effect seen in face-to-face review settings than in virtual settings, potentially due to the level of engagement. Pier, Raclaw, Kaatz, Brauer, Carnes, Nathan, and Ford [16] note differences in the nature of collaborative discussion between face-to-face and videoconference, but find no substantive difference between panels reviewing the same grant applications. Gallo et al. [10] indicate some difference between review formats in discussion time but few variances on the average overall scientific merit score, scoring distribution, standard deviation, reviewer demographics, or inter-rater reliability.

Trust is important for a successful peer review generally, and is an element of interaction most often discussed in terms of the review format (face-to-face or online). Carpenter et al. [1] indicated an important difference between face-to-face (virtual or in person) and teleconference is the level of trust between reviewers. Trust is formed via the social cues picked up on when faces can be seen [31], and on the socializing that occurs during breaks. Lavery and Zou [50] suggest that trust is built by direct, face-to-face interaction; as trust becomes deeper, through increased interaction, the result is higher quality output. Venkatraman [15] noted that virtual reviews may cause young investigators to experience a loss of trust. Driskell, Radtke, and Salas [57] indicated that teams using video conferencing took longer to establish trust, and Zheng, Veinott, Box, Olson, and Olson [58] argue that trust is highest when people meet face-to-face.

The review of literature covered skills, training, and format as articulated in funding agency reports and studied by researchers. Towne et al. [9], Coveney et al. [12], Mow [19], Abdoul et al. [32], and The British Academy [39] described training elements offered by funding agencies from which the necessary skills can be extrapolated. However, only Porter [22], Member [23], and Guilford [45] have directly discussed the skills reviewers need to be effective peer reviewers.

## Methods

### Ethics statement

Participation in this exploratory study was voluntary. Interviewees’ anonymity and confidentiality was guaranteed, and they gave their verbalconsent to be interviewed and recorded. Upon securing consent, participants were read the information statement on the study’s objectives. Survey participants were provided an information sheet once they clicked on the survey link and indicated their consent by clicking the “begin survey” button. This research and the methods described below were approved by the Oak Ridge Site-wide Institutional Review Board (FWA 00005031) ORAU000512 and complied with the terms and conditions of the LinkedIn website from which some data were collected.

### Data collection and analysis

To explore the research questions concerning peer review skills, review panel format, and contributory learning activities, data were collected in two stages: 1) interviews, and 2) surveys. This section covers the collection and analysis for each stage.

#### Interviews

*Data collection*. Telephone interviewers were held with seven experienced peer review program officers (or program managers) and five expert peer review panelists identified via the authors’ professional contacts in natural, physical, and information sciences. Interview participants’ informed consent was obtained in writing (email) prior to participation and confirmed verbally during each interview. Each session consisted of a primary interviewer, a secondary interviewer, a note taker, and the interviewee. Interviews lasted approximately 30–60 minutes and were recorded, with permission, in order to verify and clarify notes.

The researcers designed a semi-structured interview protocol consisting of five questions. The objectives of the questions were to maintain a high-level, open format that allowed interviewees to discuss their experiences in as much detail as desired, to avoid undue limitations and biases on our part, and to focus on key themes discovered through data saturation (repeated skills). An icebreaker question focused on participants’ experience as either a program officer or peer reviewer of research grants or proposals. Three questions followed asking interviewees to identify the skills or traits descriptive of the best peer reviewers, how participation in face-to-face and/or virtual panels develops peer review skills, and whether—and how—the skills needed varied based on review format. Lastly, participants were given the opportunity to add statements related to the “skills or traits needed for successful panel reviews.” The use of a semi-structured format allowed for follow up questions from the interviewers and interviewees.

**Fig 1:**
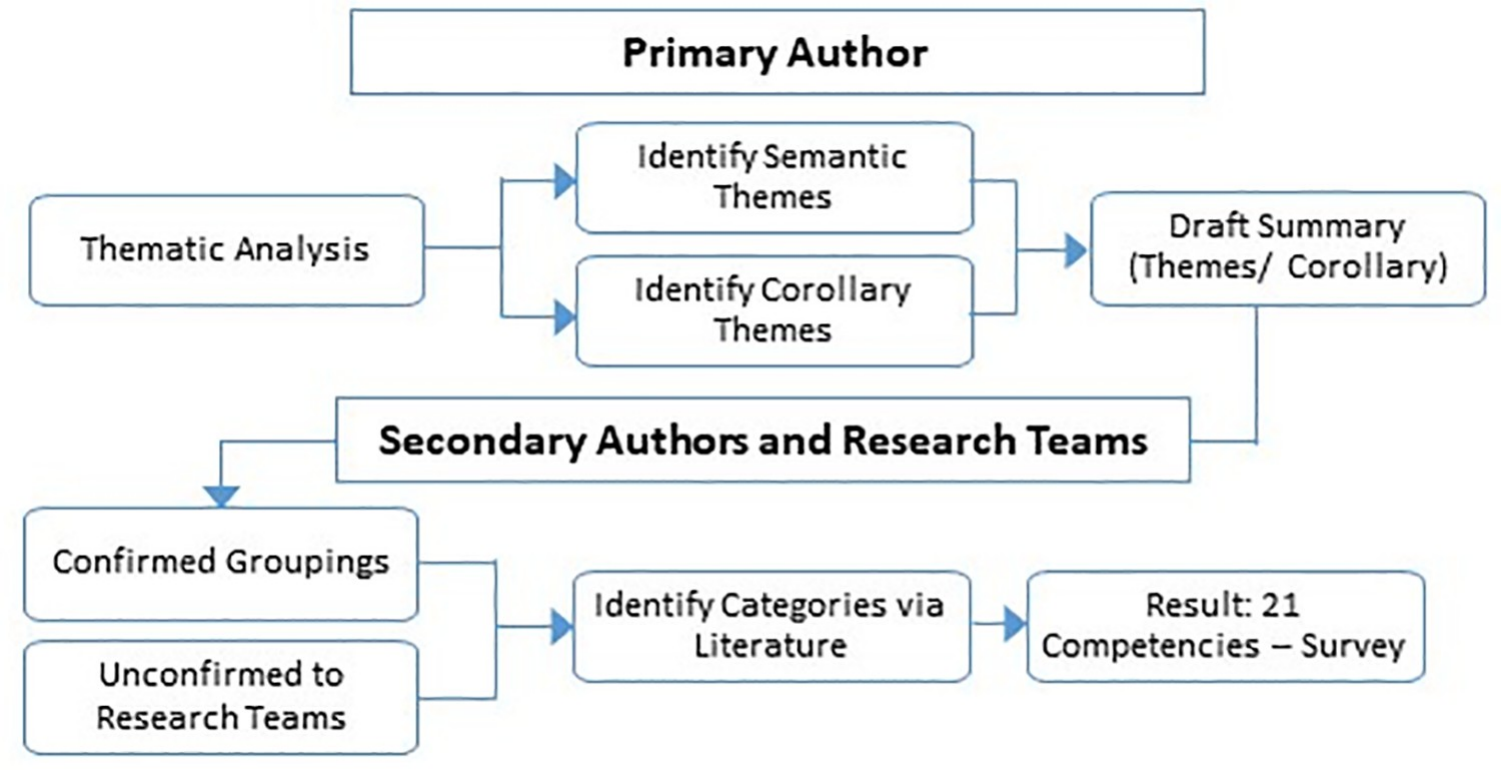
Data analysis process—Interviews to survey.

*Data analysis*. Interview notes were analyzed following the thematic analysis methods described by Braun and Clarke [59]. Using the interview questions as an initial guide, and Braun and Clarke’s [59] theoretical thematic analysis (Fig 1), or top down approach, the primary author first reviewed all responses to each question for semantic themes (repeated patterns that were important or interesting) within the “explicit or surface meaning” of the responses [60]. This was followed both by further semantic thematic analysis across questions and a deeper dive looking for latent themes [59] arising from additional points raised by participants.

The primary author then drafted an analytical summary of the themes identified, as well as the corollary findings, which were not considered thematic. This was reviewed by the research team via discussion to reach a consensus on the themes. Similar skills were grouped into larger categories using literature in social sciences. Where skills could be placed in multiple categories, i.e. a skill fell in both communicating and listening, decisions were made by the researchers in consultation with other research teams in our organization. The result was 21 elements we call competencies.

#### Survey

*Data collection*. Following analysis of interview data, an online survey was developed based on the themes identified and respondents were recruited using two strategies–a LinkedIn campaign and a purposive sampling method [61]. The population was limited to those in physical and natural sciences and potential participants were filtered using the following criteria: worked in physics, chemistry, math, computer science, astronomy, biology, engineering, environmental science, nuclear engineering, or materials science; held a doctorate; worked in the U.S.; and contained the key phrases “panel review”, “peer review”, or “grant review.” Using LinkedIn’s advertising and InMail tools, our survey link reached 8768 members who received the InMail message and/or the advertisement; 87 of those clicked on the link to the survey.

Due to the initially small number of respondents to the LinkedIn method, the research team randomly selected 15 R1 Research Universities from the 2018 Carnegie Classification list. R1 universities are research intensive, therefore this group has a higher likelihood of faculty being engaged in grant or research reviews. From each of the fifteen selected universities, five faculty members were randomly identified from each of the 10 disciplines targeted, or an equivalent number from the school’s available qualifying disciplines; 50 faculty per school. The researchers gathered all faculty member email addresses and rank information from university directory listings. From the compiled list of all faculty in a department who met the inclusion criteria, 748 faculty members were sent personal email invitations with a link to the survey. Two of the identified contacts were administrative staff rather than faculty and therefore did not receive email invitations.

Upon accessing the survey, all survey respondents’ were first shown an informed consent document outlining the purpose of the study, risks and benefits, and promise of anonymity. The form stated “By clicking ‘next’ below you are confirming and accepting the Informed Consent and agreeing to participate in this research.”

We confirmed our sample by asking potential participants two questions: Is the United States your country of residence? and Have you served as a review panelist for research proposal or grant proposal decisions? Those that responded affirmatively to these two question then answered two additional questions (perception, improvement) related to the 21 measurable panel review competencies:

- Subject matter expertise
- Familiarity with the peer review process
- Broad scientific understanding
- Knowledge of specific agencies’ peer review process
- Preparedness
- Impartiality
- Analytical thinking
- Openness to other opinions
- Clear and concise writing
- Active listening
- Open to novel ideas
- Sensitivity towards bias
- Confidence in one’s opinion
- Put proposed research into context
- Articulate ideas clearly
- Sustain attention
- Interpret body language
- Build rapport
- Redirect conversation
- Stay on topic
- Collegially disagree

Specifically, respondents rated their perception of the relative importance of the 21 competencies “to being an effective review panelist” using a five-point Likert scale (1 = Strongly disagree; 2 = Disagree; 3 = Neutral; 4 = Agree; 5 = Strongly agree). They were also asked to indicate which review format “best helps develop or improve each of these competencies.” Response choices included (1) Improved more by virtual participation, (2) Improved equally by virtual or Face-to-Face participation, (3) Improved more by Face-to-Face participation, and (4) Not improved by either format.

Respondents were then asked the extent to which 13 activities, created by the authors directly from interview responses, helped improve their overall competencies as a peer reviewer of funding proposals. The 13 activities were:

- Observation of other panelists
- Listening to panelists make arguments
- Sharing my thoughts during discussions
- Being the chair / responsible for running a discussion
- Casual discussions with senior colleagues
- Reading reviews of my own research proposals
- Being mentored by colleagues experienced in panel reviews
- Mentoring others concerning participation in panel reviews
- Serving as a peer reviewer of manuscripts for publication
- Participating on more than one panel
- Writing / submitting research proposals myself
- Academic training (e.g. graduate programs, workshops)
- Training / instructions from funding agencies

Response choices included (1) Did not improve, (2) Minimally improved, (3) Somewhat improved, (4) Strongly improved, or (5) I have not experienced this. Lastly, the survey included questions about respondents’ background (i.e., career stage, field of study, degree type), review experience (i.e., number of face-to-face and virtual panels, sponsoring agencies), and demographics (i.e., gender, age group).

*Data analysis*. Basic descriptive statistics were calculated in RStudio and Excel using the survey data.

## Results

Findings are discussed by topic based upon the research questions beginning with Skill definitions. Each topic (skill definitions, respondent characteristics, perceived importance of reviewer skills, panel format, and how reviewer skills are developed) is addressed individually. Because program officers and peer reviewers may have different perspectives on peer review and the skills necessary, we have separated out the interview results from the survey results. Our objective was not to compare these groups, rather to utilize the interviews to formulate the survey and utilize the survey to illuminate the perspectives of grants peer reviewers. We consider organizing this section by topic to be most appropriate for readability and clarity.

### Respondent characteristics

#### Interviews

Interviews were conducted with seven program officers and five review panelists who represent a collective 206 years of peer review experience. The seven program officers currently or previously work(ed) for four organizations including three U.S. federal agencies (Department of Energy, National Institutes of Health, National Science Foundation, and United States Geological Survey). The five review panelists were all PhD-level academic researchers and subject matter experts in science and technology fields. Collectively the interviewees had served as panelists for seven U.S. federal agencies and numerous additional agencies, organizations, and governments on hundreds of panels in multiple formats. The program officers had all been with a grant funding agency for at least 10 years and the panel reviewers all had at least 30 instances of serving on a panel in either face-to-face or online formats, many also had experience with blended formats of grant reviews.

#### Survey

Overall, 61 people started the survey. Ten of the responses were excluded from the analyses because (1) the respondent had never participated in a research or grant proposal review or (2) completed two or fewer survey items, resulting in a total analytic sample of 51 survey responses, with only 44 providing demographic information. Due to the anonymous nature of the survey collection, comparing the characteristics of responders versus non-responders, even for those who received individual emails (university) is impossible. The researchers are unable to estimate the characteristics of the over 8000 people who were contacted through the LinkedIn campaign as this information is not provided by LinkedIn. However, in order to provide context for the survey results and the potential biases in our analytic sample, we have described the gender, status, and field of study for the 748 faculty members who received personal invitations as compared to the self-reported gender, career stage, and field of study for the survey respondents (see Table 1).

**Table 1:** Description of initial sample^a^ and analytic sample of survey respondents.

	Initial Sample	Respondents
***Career Stage***^b^		
Early	11%	9%
Mid	26%	33%
Senior	61%	56%
Other	2%	--
*N*	748	44
***Gender***^c^		
Female	30%	27%
Male	65%	64%
Other	5%	8%
*N*	748	45
***Program***		
Physics	8%	24%
Engineering	20%	18%
Chemistry	11%	13%
Materials science	9%	13%
Computer science	11%	9%
Biology	9%	7%
Environmental Science	7%	4%
Mathematics	15%	0%
Other	10%	11%
*N*	748	45

^a^ Initial sample includes 748 faculty members randomly selected from 15 R1 research universities. The analytic sample includes all survey respondents. These groups are not mutually exclusive.

^b^ For the initial sample, assistant professors were categorized as early career, associate professors as mid-career, and full professors as senior. Lecturers and researchers were classified as other.

^c^ Gender in the initial sample was visually determined by the author and is therefore an approximation.

To insure anonymity and focus solely on overall perspectives the researchers only collected career stage (early = 1–10 years, mid = 11–20 years, senior = 21+ years), gender, and academic program demographics. Of those who provided demographic information (n = 44), the majority were male (64%) and senior researchers (56%), 33% were mid-career and 9% were early career researchers. The majority of respondents were in Physics; interestingly there were no responses from Mathematics scholars. The survey respondents’ age distribution was relatively similar, 32% (n = 14) were between the ages of 45 and 54, 27% (n = 12) between the ages of 55 and 64, and 25% (n = 11) were between the ages of 25 and 44 years.

Survey respondents had served on hundreds of panels in multiple formats. Table 2 illustrates the distribution of agencies for whom participants had reviewed and the frequency of review for each. More than 80% of reviewers had served on review panels for the National Science Foundation, more than half on panels for the Department of Energy, and one-fifth for the National Institutes of Health.

**Table 2:** Frequency of respondents’ panel review participation by agency.

Agencies	n	% (n = 45)
NSF (National Science Foundation)	37	82%
DOE (Department of Energy)	24	53%
NIH (National Institutes of Health)	9	20%
DOD (Department of Defense)	8	18%
NASA (National Aeronautics and Space Administration)	6	13%
International Agency(ies)	5	11%
USDA (United States Department of Agriculture)	5	11%
DHS (Department of Homeland Security)	2	4%
EPA (Environmental Protection Agency)	2	4%
CDC (Centers for Disease Control & Prevention)	1	2%
NIST (National Institute of Standards and Technology)	1	2%
Other*	12	27%

*Of those who selected other and provided additional information, the following meaningful responses were recorded: National Historical Publications and Records Commission (NHPRC), Institute for Museum and Library Services (IMLS), National Endowment for the Humanities (NEH), Social Sciences and Humanities Research Council (SSHRC),

Council on Library and Information Resources (CLIR), National Park Service (NPS), National Energy Technology Laboratory (NETL), American Heart Association (AHA), Juvenile Diabetes Research Foundation (JDRF), Research Corporation, Kaufman Foundation, Beckman Foundation, Welch Foundation, Internal grant review at my institution, Smithsonian, Soros Foundation, Greek funding agencies, Czechoslovakian funding reviews, Austrian Science Foundation, European Agencies, and “review committees for several foreign institutions”.

Almost all survey respondents had participated in both face-to-face and virtual panel formats. Ninety-three percent (n = 42) indicated participation in at least one face-to-face panel and 89% (n = 40) in at least one virtual panel. Very few respondents had more experience in one format as compared to the other. Six respondents participated in 26 or more face-to-face review panels, however, no respondents participated in more than 25 virtual panels.

### Skill definitions

Definitions of skills were taken from interviews, thus there is no corresponding survey element in this topic. Interviewees indicated that reviewer skill was impacted by how often one participated in panel reviews, the agency sponsoring the panel, panel format, and the career stage when panel participation occurs. They also noted that the purpose and nature of panel reviews differed among agencies. For example, some funding agencies require panelists to reach consensus in their recommendations whereas others allow disagreement. In some cases, the final funding decision rests with the panel, in other cases the panelists make recommendations to the agency who then determine the final funding decision. These differences impact the skills required for, and developed within, peer review panels.

Using thematic analysis of the interview notes, the researchers constructed 12 themes that describe the capabilities of the best peer reviewers for reviews of research funding. The themes were Subject Matter Expertise, Broad Scientific Understanding, Impartiality, Time Management / Being Prepared, Attending to the Purpose, Understanding the Purpose and Role of Peer Review, Communication Skills, Technical Adeptness, Analytical Thinking, Interpersonal / Social Skills, Open Mindedness and Trust in Self, and Diversity.

Subject Matter Expertise and Broad Scientific Understanding were the most commonly mentioned and thought to play off one another in the sense that although deep subject matter expertise is almost universally required, without the ability to understand research in context it is significantly less useful. As one interviewee stated, both “broad and deep knowledge of subject areas” are important.

Fairness and sensitivity towards, avoidance of, and the ability to mitigate, bias and/or conflicts of interests are described as Impartiality. Time Management/Being Prepared were expressed as managing one’s time so as to complete tasks as expected and be fully prepared to participate at the agreed upon time. Attending to the Purpose of the review by reading not just the proposals, but the request for proposals, following directions, conforming to the expected process, and ensuring the criteria for decision-making are followed was also considered vital. One reviewer summarized this by stating, “Take it seriously. The purpose is to (1) find and fund the best science, and (2) help develop future scientists.”

Understanding the Purpose and Role of Peer Review was less frequently, but still distinctly, noted as compared to other skills. It refers to the concept of peer review in general (as opposed to Attending to the Purpose of a specific review effort), and its importance to, and role in, the scientific enterprise. In other words, to be effective, panelists must buy in to the concept of peer review of research.

Communication Skills included speaking, writing, and listening and were nearly universally discussed. English proficiency and the ability to synthesize thoughts clearly and concisely in writing and/or verbally, were included. It was also noted “being an effective communicator face-to-face is different than being an effective communicator virtually.”

Technical Adeptness was described only with respect to virtual review formats and comprised the ability to sustain appropriate audio levels and clarity, internet connections, and camera placement. Analytical Thinking referred to the ability to complete an evaluative analysis by weighing the individual and comparative merit of proposals. Reviewers need to be able to “identify strengths and weaknesses, judge relevance, and critically evaluate the contribution to science.”

Interpersonal / Social Skills are important to the review panel process and include listening to other reviewers, interacting respectfully, managing interactions, and engaging “with a spirit of contribution and improvement as opposed to apathy or negativity.” One interviewee summarized this by noting that it is “interpersonal relationships and abilities” that “distinguish panel reviews from individual reviews; panel review success is the combination of technical expertise and interpersonal relationships and abilities.”

The fact that panelists need to be skilled in “the delicate balance between being open minded enough to be willing to change one’s mind when appropriate, yet confident enough in one’s opinions and knowledge to stick to what one thinks when important” encapsulates Open Mindedness and Trust in Self. Diversity was described as a trait of a panel rather than a reviewer. Panels with institutional, demographic, and scientific diversity were considered more balanced and therefore better by both review panelist and program officer interviewees.

### Perceived importance of reviewer skills

To assess the relative importance of reviewer skills among a larger group of stakeholders, the authors developed a list of 21 measurable competencies based on the reviewer skills identified and described in the interviews, the panelist literature [23,30,31], and the professional skills literature [36,37,62].

#### Interviews

Respondents suggested important competencies in the open-ended questions, including “know who the other members are and their background”, “understand the politics of the funding agency, competing research groups, etc.,” and “understand goals for the funding agencies.” One interviewee stated, on the importance of interpersonal skills, the panel “is a team, meaning ‘you have to play well with others’.” Two comments on the concept of consensus were voiced (1) “there has to be room for vigorous disagreement as there are questions where consensus has not yet emerged”, and (2) “consensus is not the goal; fair and unbiased evaluation against a consistent set of criteria and standards is the goal.” One interviewee added that “Many of [the competencies] have increased importance as panels begin to move to remote panel reviews [using] teleconference or video conference where the ability to stay on topic and professionally direct the conversation is vital.”

#### Survey

**Fig 2:**
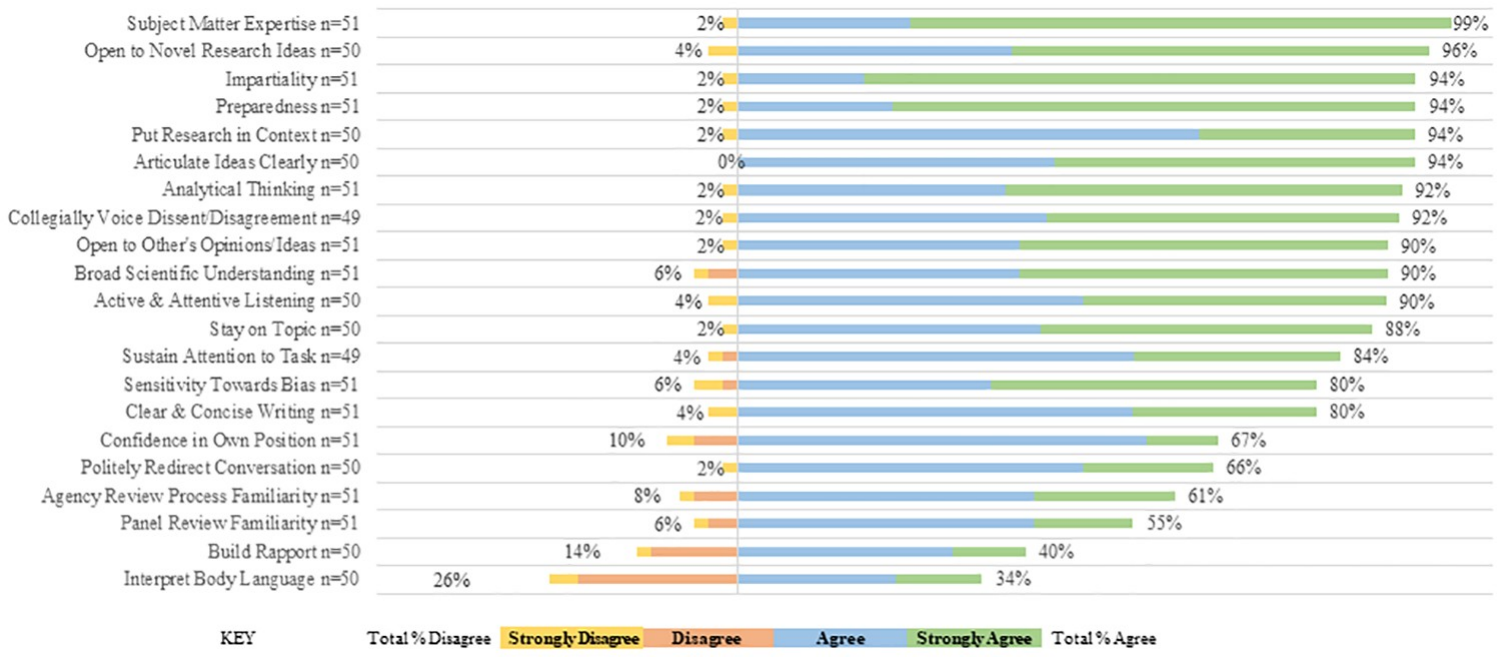
Perceived importance of competencies.

From the competencies mentioned by the interviewees, survey respondents were asked about the importance of each competency for panelists. Fig 2 gives the frequency of survey responses regarding the importance of each competency. For all but two competencies (Build Rapport and Interpret Body Language), more than 50% of survey respondents agreed or strongly agreed that the competency was important for being an effective review panelist.For more than half of all of the included competencies, 90% or more respondents indicated they were important. Rising to the top of the list were: Subject Matter Expertise, Openness to Novel Research Ideas, Impartiality, Being Prepared, ability to Articulate Ideas Clearly, and ability to Put Research Into Context, all of which were endorsed by 94% or more of survey respondents. Respondents indicated that familiarity with processes (agency review process, panel review experience), rapport building, and the ability to interpret body language were less important.

### Panel format

This exploratory study attempted to focus on two panel formats–face-to-face and virtual. This choice was meant to determine if there are any skills required in one format but not in another. All skills were considered necessary in both formats, except Technical Adeptness.

#### Interviews

Concerning the relationship between technology use within review panels and panelist skill, interviewees felt the skills needed in each setting were similar, but that virtual participation was more difficult than face-to-face. They noted virtual panels require “more sustained attention, better technical skills, and more developed interpersonal and communication skills, such as higher level listening skills.”

#### Survey

**Fig 3:**
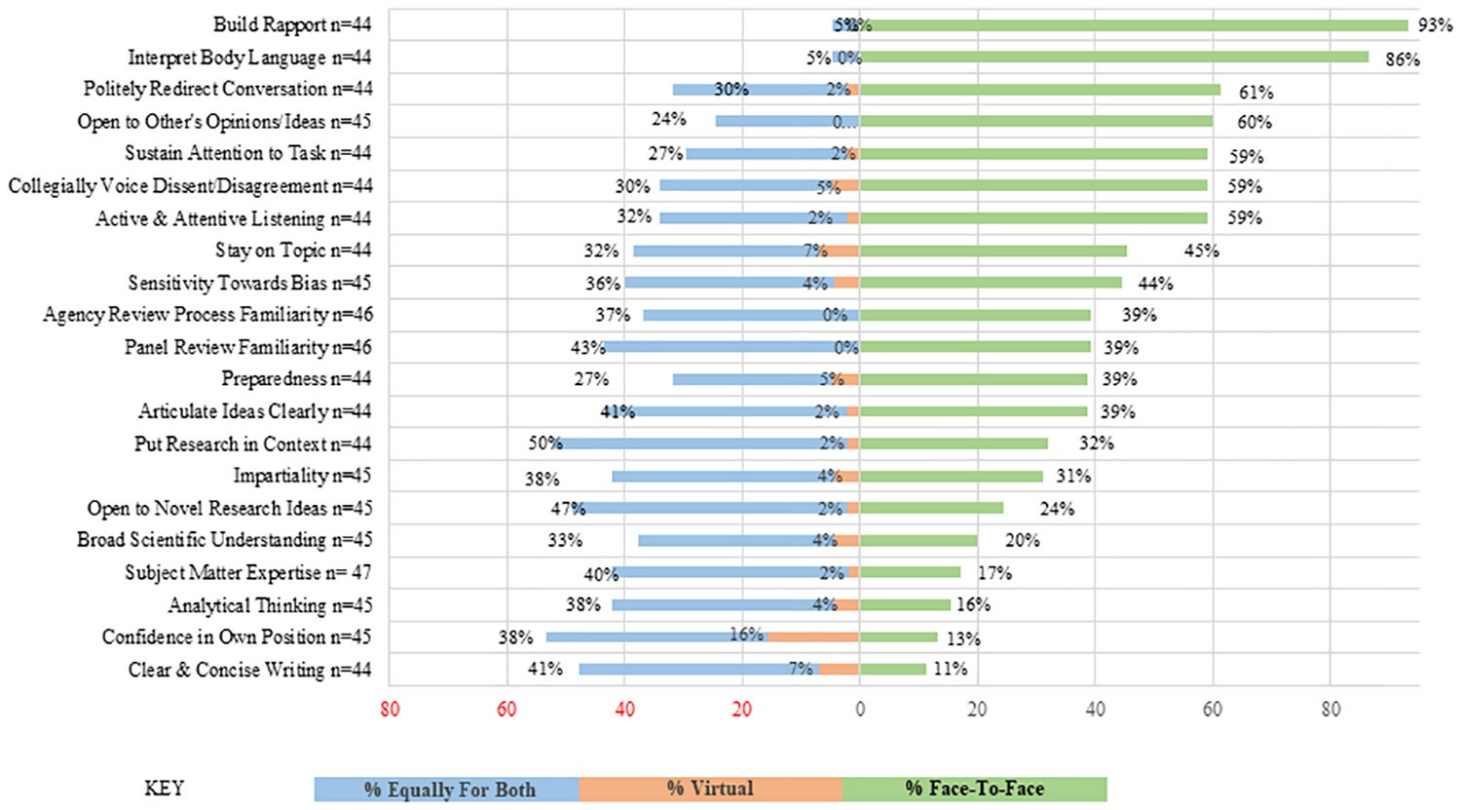
Ability to develop competencies in different formats.

Survey respondents were asked to indicate which panel setting (virtual or face-to-face) best helps develop or improve each competency (Fig 3). They could also indicate whether the competency was equally improved by virtual or face-to-face participation formats, or was not improved by either format. Largely, if a preference was indicated, respondents noted the competencies were improved more by face-to-face participation. Very few respondents indicated that competencies would be better improved through participation in a virtual review panel. Respondents indicated seven of the 21 competencies were more improved by face-to-face participation: Build Rapport (93%), Interpret Body Language (86%), Politely Redirect Conversation (61%), Open to Others’ Opinions/Ideas (60%), Active Listening (59%), Politely Disagree (59%), and Sustain Attention to Task (59%). Forty-percent or more of the respondents indicated that Panel Review Familiarity (43%), Articulate Ideas Clearly (41%), Put Research in Context (50%), Openness to Novel Ideas (47%), and Subject Matter Expertise (40%) were equally likely to be improved in either setting. Confidence in one’s own position was the only competency for which greater than 10% of survey respondents indicated it would be more likely to be improved in a virtual setting.

### How panelist skills develop

Given certain skills were considered important and little to no training on those skills was provided, the researchers asked about activities that may help develop the 21 competencies.

#### Interviews

Interviewees generally stated peer review skills were needed for effective participation in panel reviews but were not necessarily developed through participation. For example, one interviewee stated, “I don’t know if [either format] develops them, as much as takes advantage of them … I think you bring a lot of the skills with you …” Another said participation on a panel allowed one to “gain an appreciation” for the skills needed to be an effective panel reviewer, but overall, interviewees shared the sentiment that “most of the time… [program officers] think I have the skills already.”

When considering skill development specifically, interviewees noted participation in virtual panels made development more difficult. For example, one interviewee commented “skills develop to a lesser degree in virtual settings” while another hazarded that “perhaps skills develop only half as well as in face-to-face settings” [63]. Virtual settings were considered less engaging for participants and required more effort from the reviewers to pay attention and not get distracted. In the virtual setting, the management of the panel (how it is run) was seen to be as important as the panel itself. One interviewee stated program officers must “make sure to cue participants in to what is happening, be aware of noises like shuffling of papers and scraping of chairs, and be deliberate about capturing results, timelines, breaks, etc.” Because there are no cues from which to read these things (in online formats) everything must be explicitly handled.

Modeling (observation of others/mentoring others) and “on the job training” during panel participation were two ways interviewees described for reviewers to develop their skills. Having the process of reviewing grant proposals modelled by more experienced reviewers includes (1) carefully listening to what is going on, whether “to what it is that other people think is important in a proposal” or “to someone make an argument, trying to understand the strengths and weaknesses,” (2) learning “directly from other panelists as to how they make decisions/judgements by having small side conversations with a more experienced panelist,” and (3) having “[one’s own proposals] reviewed and read[ing] the comments.” One interviewee noted that reviewing manuscripts can develop several of the required skills such as “being able to judge what is required to successfully complete the research, whether the question has merit, the methods support it, etc.” However, this individual also noted that while there are some similarities in the skills required in reviewing manuscripts and grants, making a funding decision is different than accepting or rejecting a manuscript and that there are different criteria involved for awarding money. With regard to modelling the process of peer review for others, rather than having it modelled for yourself, one interviewee noted the benefit of exposing junior faculty to the process of writing proposals via mentoring.

There was disagreement as to the extent to which formal training concerning how to review research proposals was occurring and the utility of what was offered. Some felt there was “an awareness of the need” at certain agencies with those agencies providing instructions and time for reviewers to ask questions before beginning the process, while the opposing view was “For better or worse, I would say we don’t train [reviewers] at all. There’s no formal process. We provide them with guidance.”

#### Survey

Survey respondents were asked to indicate whether specific experiences, described by the interviewees, improved their competencies. The majority had experienced all 13 activities, with the fewest participants having served as a chair (61%), received mentorship from colleagues with panel experience (70%), and mentored others (76%). However, of those who had experienced the activities, 96% felt that Being Chair either somewhat or strongly improved their competencies. Similarly, Listening to Panelists Make Arguments, Sharing Thoughts during Discussions, and Serving as a Peer Reviewer for Publications were also highly endorsed by survey respondents. Training materials and resources from academic institutions or funding agencies were viewed as the least helpful, though 50% or more of respondents felt the activities somewhat or strongly improved their competencies as a reviewer.

Helpful experiences listed by survey participants included conflict resolution courses, leading technical discussions in the field with both expert colleagues and professional users of that information, and a good program officer/chair who sets clear expectations at the beginning and reminds panelists as needed. Finally, diversity of reviewers’ backgrounds on a panel was considered helpful in broadening ones thinking.

## Discussion

This exploratory mixed-methods study fills a gap in the peer review literature concerning the skills needed by peer reviewers of research funding proposals. Notably peer review skills are considered professional skills. The literature suggests such skills are important for scientists. Additionally, the skills identified by those interviewed demonstrate a connection between the skills of effective peer reviewers and the professional skills needed by successful scientists. For example, the technical aptitude identified as necessary for effective participation in virtual reviews is an extension of the technical aptitude already seen as required to conduct modern science. In addition, despite the relatively small survey sample, survey results yield insights concerning the relative importance of the review panelist skills that were identified and the activities by which these skills are best developed.

The identified skill of Impartiality reflects recognized professional skills such as best practices and critical thinking. As Davis, Conner, and Shapard [63] state, “time management, following directions, attending to purpose, being able to communicate, and getting along with others are all the ‘interpersonal, human, people or behavioral skills needed to apply technical skills and knowledge in the workplace,” in this case, in the scientific workplace. Despite this, understanding the purpose and role, as well as being adept at participating in peer review, is critical for professional scientists. What was uncovered in this exploration was that these peer review/professional skills are either not included in scientists’ training, or, when they are, are provided without context.

Little has been written in the peer review literature about the skills needed for effective panel review participation and how such skills might be developed has received less discussion. This makes it difficult to interpret our findings in the context of extant research. Interviewees described two ways they had developed their panel review skills: (1) modeling by others, and (2) “doing it”. These two methods are supported by the fact that Being the Chair/Responsible for Running a Discussion was the highest rated skill development activity among survey respondents. Listening to Panelists Make Arguments and Participating in More than One Panel were considered significantly more likely to improve panelist competencies than the overall likelihood for any skill improvement activity.

Training Instructions from Funding Agencies and Academic Training were considered the least able to improve panelists’ competencies. Is this because training was experienced by a smaller number of respondents and therefore its utility was minimal, or is the existing training ineffective? Moreover, if the training that is offered does not improve panelist competencies, what activities or experiences would improve these? This exploration suggests that more examination and evaluation of the training and instruction offered by funding agencies needs to be conducted, and supports the literature that calls for more professional skills training for graduate students.

Face-to-face panel format is considered superior for improving several of the most important panelist skills; however, other important skills were deemed by respondents as equally improved by either format. The importance of possessing the necessary skills prior to, or developing those skills via participation in, a panel review was inconclusive. Therefore, making clear conclusions about skill improvement based on panel format is difficult. Respondents were not asked, and did not volunteer, why they considered a particular competency more improved by one format than another. Further investigation in general, as well as examining which elements of face-to-face formats assist reviewers in improving their skills, is warranted.

While some of the peer review literature debates the setting and the effect of technology on panel reviews, little discussion was uncovered concerning differences in the skills needed in the two settings. Impacts to communication were the exception; however, the discussion was focused on the quantity and quality of communication in different settings, not on communication as a skill. The overall sentiment that virtual participation is more difficult than face-to-face participation, supported by our finding that virtual participation did not improve any competencies more than face-to-face participation, emphasizes the importance of communication skills in both formats.

The small sample size for this study suggests our findings cannot be universally applied or generalized, however, they do provide a hint at potential interpersonal aspects of peer review activities that could be studied more. We intentionally focused on Research 1 (R1) universities in the United States in 10 fields within the physical and natural sciences. The perspectives and opinions we collected are interesting but do not capture the breadth of peer review for research funding universally. Studies by Mow [19] comparing social and natural sciences, or Coveney et al. [12] comparing the Basic Science and Public Health reviewers, indicates that research cultures are different. Those differences should be explored more thoroughly to uncover different skills, interpretations, or emphasis based on the research culture. Understanding the influence of research culture on the training, interpretation, and use of particular skills will become more important if, as Abdoul et al. [32] suggest, peer reviews become more transparent or as panels become multi-disciplinary.

There are numerous disciplines that were not considered in this exploratory study. Our goal was to focus narrowly on peer review in our organization’s main fields, science and technology. However, a larger and broader sample would enable researchers to explore the relationship between perceptions of the degree to which activities improve competencies and their experience with the activities themselves. While our sample included early career respondents, a much large sample across all career stages would illuminate the relationship between panelist review skills, review skill development, and career stage.

The manner in which panel reviewers acquire the necessary skills needs further exploration as well. Towne et al. [9], Coveney et al. [12], Mow [19], and Abdoul et al. [32] examined funding agency materials, reviewer training documents, and funding opportunity guidelines. All sources examined in this exploration (literature, interviews, and survey responses) indicated there is a lack of skills preparation in graduate school for effective review participation. An interesting investigation would be to explore the methods of learning utilized by peer reviewers, outside of formal pedagogical settings. Examining funding agency documentation, graduate school training seminars, and non-pedagogical learning practices will lend additional information concerning the type of training to develop and the method by which to provide it to new and potential reviewers.

The important role of the moderator or chair in panel success, particularly in settings relying upon technology for panelist participation, was noted by both interviewees and survey respondents. In fact, the opportunity to be the chair of, or lead, a review panel was rated the most important activity in improving panelists’ skills. While not related to issues of technology, interview respondents in Coveney et al.’s [12] study noted the important role of the chair in ensuring “the group kept on task and dealt with proposals fairly” [15]. Together, these results suggest lines of inquiry concerning the moderator are necessary. Such investigations could focus on interventions that offer leadership opportunities to a broader proportion of reviewers, or studies on what makes the role of the moderator so useful in developing panelist skills.

Results also indicate face-to-face panels are preferred as a way to improve panelist skills, however, they do not suggest why respondents indicated a particular competency was more improved by one format than the other. Therefore, additional research is proposed to determine the characteristics of different panel formats that assist reviewers in improving their skills. Overall, an increased focus on peer review panelists’ skills and their development is not only warranted but serves to ensure that peer review of research submitted for funding is not only fundamental to science, but sustainable as well.

## Supporting information

S1 Appendix Interview protocol. (DOCX)

S2 Appendix Interview data. (DOCX)

S3 Appendix Survey instrument. (PDF)

S4 Appendix Survey data. (XLSX)
