# Supplementary material for: What makes an effective grants peer reviewer? An exploratory study of the necessary skills
Source: PLoS One. 2020 May 13;15(5):e0232327. doi: 10.1371/journal.pone.0232327 (PMC7219739; doi:10.1371/journal.pone.0232327)
Supplement: S1 Appendix — (DOCX) [file pone.0232327.s001.docx]

RQ: How do the development and realization of peer review skills differ in different review formats?

**___ At least one day before the interview, confirm the interview with the participant, including location.**

**Introduction**

*Hi, my name is _________ and I am a __________ in the ___________ department at ORAU. I am calling for the interview on peer review of research skills. Thank you for taking the time to participate.*

*I will be conducting our interview. [Second person] is also calling in. [Second person] is also a ________ in the _____________ department at ORAU. [Second person] will be taking notes. [Second person] may also ask questions or make comments.*

*Thank you for being willing to speak with us about your views on the skills development of peer reviewers of grant proposals.*

Purpose

*As part of a study concerning peer review of research skills and how they are developed in different review formats, we are conducting interviews with experienced peer reviewers to determine what skills or traits you think are necessary for an effective reviewer to possess, and your thoughts on how these skills might be developed through participation on a panel review for grant proposals.*

Structure

*We will ask you 5 questions about your perspective. We will utilize the results of these interviews to build a survey that will be given to professionals who have participated as grant panel reviewers in face-to-face and/or online formats. This interview should only take between 15-20 minutes.*

*We would like to record this conversation for accuracy and verification purposes. The recordings will not be transcribed and they will be deleted upon development of our survey instrument. May we have your permission to record this interview? (Yes or No)*

*This interview poses minimal to no risk to you and you may decline to continue at any time. Are you still willing to participate in this interview? (Yes or No)*

*---------------------------------------------------------------------------------------------*

Turn on recorder.

*OK, I have turned on the recorder. This is an interview of INTERVIEWEE by INTERVIEWER on DATE at TIME. [Second person] is also participating, and __________ is taking notes. For the record, can you please state that I have permission to record this conversation?*

Interview Questions:

Q1. Icebreaker –

To get us started, please describe your experience as a program officer / peer reviewer for peer reviewer of research grants or proposals.

Q2. OK. Thank you.

What skills do you feel describe the best peer reviewers?

- 1. Clarification: What skills or traits describe an expert reviewer?

Q3. How does participation in face-to-face review panels develop these skills?

1. Rephrase: How does participation in face-to-face review panels develop the skills you noted?

Q4. How does participation in online (or virtual) review panels develop these skills? These include teleconference or videoconference settings.

Q5. Are there skills that are critical in one format, but not needed in another?

Q6. Is there anything else you would like to share with us related to skills or traits needed for successful panel reviews?

Thank you for your time and input.
