# Supplementary material for: What makes an effective grants peer reviewer? An exploratory study of the necessary skills
Source: PLoS One. 2020 May 13;15(5):e0232327. doi: 10.1371/journal.pone.0232327 (PMC7219739; doi:10.1371/journal.pone.0232327)
Supplement: S2 Appendix — (DOCX) [file pone.0232327.s002.docx]

**PO1 – 04/15/2018**

PO1 - Q1. Experience

Exp: 10 years; one program has 2 panels per year, averaging 12 people per panel. Second program preliminary 400-500 per year proposals, 100 selected. Seen online and assisted purely online and blended formats.

PO1 - Q2. Skills

Content knowledge, listening skills (especially interdisciplinary panels, range poor to excellent), important to understand different perspectives, well-prepared (read and written your review, ready to discuss); open minded; articulate rationale; flexible (what they may not have considered and make adjustments); respect for other opinions; ability to interact and to communicate (not threatening, condescending, pretentious)

PO1 - Q3. F2F

Takes advantage of skills rather than utilize them; Learn quickly how to explain oneself; Socializing with other panelists, getting to know them personally helps to interact professionally; Learn how to evaluate grants; Hard to learn on-the-fly, things move quickly; Bring skills with them

PO1 - Q4. Online

Online more difficult; Not as much opportunity for conversation in online; Harder to get interaction; Technological problems

Aware of skills they do not possess – how they get them – in graduate school – depends on the program; nice to have a program to get them to present to non-experts in the field. If institution promotes being an academic then they train students.

PO1 - Q5. Critical

Have not found that either [format] is better; Do well in one format you will do well in the other; More ___ in virtual because lack of interpersonal interaction (speaking loud enough, speaking into the microphone, need technical skills to participate)

PO1 - Q6. Other/ Additional

Challenging to think about – putting together a panel and trying to balance the group. It is harder when working off of CVs, don’t know how they will be as a panelist; [PO1 uses] previous interactions or asks other people

**PO2 – 04/12/18**

PO2 - Q1. Experience

Spent 8 years as a Program Officer at [agency] in mathematical sciences; Led peer review for [agency] proposal submissions in a broad range of mathematic topics

PO2 - Q2. Skills

Ability to see research in a broader context. Funding agencies have particular goals when they release RFP - reviewers need to put the proposed research in context of announcement - broader importance to the field?; Fairness – critical able to assess research in a fair minded way, avoiding implicit or personal bias; Think analytically, needs to do an evaluative look at the research and compare to other research in the panel - assume the reviewer will evaluate multiple proposals; Good writer

PO2 - Q3. F2F

Helpful to know other people in the field, good network, know the field and what is being studied - allows individual to put research in context; Helps to interact interpersonally with others on a panel review so they can engage with others in the process, If ability to do so, will get a lot more out of the review; Ability to write evaluative comments in a critical way, not summary of proposal, [agency] already knows subject – [agency] needs someone to share strength/ weakness of proposal. Evaluation / discernment of the proposal; Intangibles - stay on point, not lead group into digressions or tangents; Sensitivity towards issues of bias and understanding broader issues of peer review; Understand process and roll of peer review in general – when is evaluation needed? What does good evaluation look like?

PO2 - Q4. Online

In person: Being exposed to the process, learn how it is supposed to work especially if see a good model in play. The best way to learn to be a good reviewer is to participate in a good review.

Virtual: Tougher. Restricted to auditory information with a touch of visual, limits how we interact. Can be difficult to set an order to speaking when online. How do you communicate virtually with a lot of people? Very little research / best practices on how to get the maximum performance on the group.

PO2 - Q5. Critical

In person: Gain knowledge in own field; Learn by watching and listening to what other people see as important in a proposal; Strengthen networking skills and expand network; Skills hardest to gain are: ability to write good evaluative analyses, having deeper understanding of broader impacts such as diversity and evaluation.

Virtual: Dependent on platform, develop skills to a lesser degree. Closer virtual is to representing F2F experience, the better the review. Video is better than telephone. Dependencies: most difficult for folks to be on a phone, especially all day. That person is not able to give 100% for a full day. Video is a little better to see the visual cues with others and a moderator to understand the structure. The moderator must be skilled at letting reviewers know when they need to be engaged

Developing close working bonds are critical. You get a deeply engaged reviewer, thinking harder on the work in front of them and they are learning more from each other that will help them come to better decisions.

PO2 - Q6. Other/Additional

Training is getting better at least at [agency]. Awareness of the need especially going to more virtual panels.

**PO3 – 04/13/2018**

PO3 - Q1. Experience

Program officer [agency], ran 8 programs, peer review for 100+ proposals. Subsequently with [agency] in [state], seen many panels and been a reviewer on many panels.

PO3 - Q2. Skills

Broad and deep knowledge in the subject area (subject expertise); Big challenge is to have a diverse panel as a PO ensure build ½ slots with women and underrepresented groups before asking the first male panelist; Diverse perspective based on race, gender, culture to have a good discussion effectively and smoothly; Concise synthesis of their thoughts and opinions; Good listeners – (if not a good listener) indicates not really being open-minded about the process

PO3 - Q3. F2F

Typically had lot of new people on panels, experience level; Would present how panel runs, logistics, what to expect, summarizing your perspective and ad hoc reviewers before you; Have experienced reviewers summarize the proposals; [agency]-lead reviewer leads all discussions, Secondary provides input as well, more experienced leaders set the tone for more junior people.

PO3 - Q4. Online

More challenging because … (personally is biased towards F2F); Participate – interruptions, doing email, etc. – it is harder to be really engaged; Having junior faculty participate remotely is a huge disservice to them – can learn more about scientific enterprise via F2F; Fractional benefit – 50% as in person; Miss facial expressions and body language - even best video technology does not do that justice; Need the absolute best technology possible – all speakers and remote participants; Must go out of the way to ensure remote participants are introduced and feel a part of the group

PO3 - Q5. Critical

Key is the ability to play in the sandbox, collegial and intellectually engaged; More challenging for remote folks to do that; Ability to focus harder for remote to effectively do; Anyone can tell [reviewers] – most learn by doing it. Those most effective are those who have done F2F and know the gravity and the process; A lot of POs are not fully cognizant of this – needs to be effectively engaged – headphones and awesome technology, webcam, etc.

PO3 - Q6. Other/Additional

Something often ignored – recognize that average panel member is extraordinarily liberal with their own research, but when join a panel they become ultra conservative; [Reviewer would say “so innovative has lower probability of success, therefore …” do not support]; [PO] has asked panelists to provide one to two names of the most far-out proposals they read; PO [job] to get reviewers to step out on a limb… [PO] learned this from a mentor PO and with others – most POs do not do this.

Sandbox – if you have one or two bad apples on a panel can cause problems; vetting reviewers before you choose – ask for names of good reviewers.

Panels – [agency] programs that are perpetual – some overlap in panelists, 2-3 years and then retired from service – 50% new blood; Team or collection of individuals on each panel develops its own personality; Starts as individuals and hope they develop as a team; Learn by observation (teaching juniors)

**PO4 – 04/13/2018**

PO4 - Q1. Experience

[Agency] PO for 10 years; second [agency] since 2009. Role – primary interface with scientific community – write solicitation, conversations, and eventual selection. Identify set of reviewers to review proposals. Panels not normal at [agency office], but do use panels sometimes – [other agency] historically uses panels - 1^st^ round proposals via panel, discussed on panel.

PO4 - Q2. Skills

Primary – how we choose peer reviewers – scientific and technical expertise; Critically evaluate proposals related to, but outside there area – relevant expertise and be able to critically evaluate; Articulate in writing

PO4 - Q3. F2F

[Agency] oral discussion that informs individual reviewers; Verbally state opinion/ viewpoint; Interact in a group (social), drives conversation forward since not asked to come to consensus; Participation will help reviewer learn by being around others; Skills of interacting in constructive way (learned skill) participating will help; Someone chairs the panel – the right person can facilitate good discussion; Junior scientists are not trained at all, the PO must provide guidance, present [agency office] expectations; Free form – not constrained by design

When putting together a panel – what’s different when choosing that versus online? Panel – broader, X proposals – diversity (institutional, breadth, gender) try to avoid overly dominant personality. Online – is targeted. Networking – not always obvious of the benefit of F2F – cost/ effort outweighs any benefits.

PO4 - Q4. Online

Never done virtual panel discussion – some in [agency office] have done this and it is becoming more common; Easier to find reviewers if use this model.

PO4 - Q5. Critical

[no response]

PO4 - Q6. Other

For large center, like [example], reverse site visits – panel does this, probe researchers; Use panels to review on-site the projects going on at lab, etc., to renew or continue on-going projects at labs – still submit individual reviews; Open-mindedness.

**PO5 – 04/16/2018**

PO5 - Q1. Experience

PO for 10+ years and manage a portfolio of research grants. Every year carry out at least one merit peer review and every few years have a panel review where reviewers travel to location to review proposals. Peer review allows [agency] to make funding recommendations and decisions.

Two classes of reviews in our [agency office] 1) mail review - select reviewers and send proposals via [online tool] to reviewers - reviewers give comments by a deadline; 2) organize a panel – depending on number of proposals – 5 reviewers attend a panel meeting around the [location] area. The review takes 1 – 1.5 days. Receive proposals prior to panel meeting and submit comments. [PO5] considers panel reviews a superior tool, only problems is they involve more travel, are more expensive, and available reviewers can be limited by who can travel.

Unlike [agency], the [agency] cannot accept a consensus report from the reviewers. Reviewers provide individual assessment and the Program Manager must interpret/evaluate the input from the reviewers. Then, the Project Officer makes funding recommendations to the office.

PO5 - Q2. Skills

Knowledgeable in the area of science under review. [Agency] try to use reviewers through reputation, publication, knowledge in the area and have done research in the area. Other issue is bias or conflict of interest. Reviewers may have collaborated with the applicants or have a well-known dispute with the work. Mitigating positive or negative bias; Past performance of the reviewer - Look at past reviewers to see how useful their details and how thoughtful their comments were; Looking for institutional balance – need a variety to avoid institutional bias; International reviewers – English language skills are required during the panel discussions; Common sense –read proposal and solicitation prior to the panel (have seen reviewers come in unprepared).

PO5 - Q3. F2F

If participated in F2F review, they understand what [agency needs], when [reviewer] develops experience [reviewer] is more useful; Reviewers learn something new from the scientific topics.

Preparation provided? Receive information prior to review (questions, link or copy of the RFP, description of what the agency wants from reviewer and what agency wants to fund). Send instructions to reviewers via email or in [online tool] that reviewers are supposed to read. Two weeks to review information and ask clarification questions prior to review.

PO5 - Q4. Online

A [agency] advisory committee which meets ~4 years recommended using more virtual panels, [in part] due to travel schedules. International reviewers [and time differences] make teleconferences difficult to schedule. [Agency] wants to explore virtual meetings more.

PO5 - Q5. Critical

Skills would be similar. Sometimes technical problems, can be distracting and difficult for the reviewers. This is weak point for virtual meeting.

PO5 - Q6. Other/ Additional

Most successful reviewers provide substantive comments. [Agency] relies on their words to measure weakness/ strengths of the proposals; Timeliness. In the mail reviews, reviewers often ask for extensions. Try to receive full comments during the panel. Some require more prompting than others; Finding reviewers can be difficult and that they don’t have enough reviewers. Would like a higher acceptance rate from reviewers.

What impacts the acceptance rate?

People funded by [agency] are more responsive as they want to be seen as helpful. They also want quality reviewers to read their own proposals. Have trouble getting those outside of the [agency] community as they don’t know [agency] very well and [agency] doesn’t know them well.

What would you want to share with a new PhD when reviewing for the first time?

Difficult to send them past reviews to get an idea of what a good (or bad) review looks like; Treat this as part of your job, even though it is a voluntary; Read the solicitation carefully; Read the proposal carefully; Don’t be afraid to contact [agency] to ask questions.

**PO6 – 4/17/18**

PO6 - Q1. Experience

PO at [agency], managed peer review process of [agency], managed a number of panels; involved in development of RFPs with [agency] (10 years) and managed panels

PO6 - Q2. Skills

Best reviewers has disciplinary knowledge of topic in proposal or paper; Buys in to peer review process as important to science; Someone willing to go into it with spirit of contribution and improvements rather than apathy or a negative perspective.

Relatively high executive function (questions asked: does the proposer have ability to do this, does this proposal support our high priorities, is this sustainable) – keep all this in mind while reviewing and keep review to a reasonable time. Scoring proposals – and ranking against each other – have to go through process for each one and keep in mind thoughts on previous one and rank on previous one.

Listen to other panelists; Prepare description (strengths weaknesses)

Junior colleague: Process of review proposal different than a paper; Will be suggested set of criteria to respond to – be sure to do that ([agency] wants strengths/ weaknesses/ impact); Be structured – conform to expectations funding agency is providing to you.

PO6 - Q3. F2F

[no response]

PO6 - Q4. Online

More panels are becoming online. Skills different – good etiquette – cannot see people’s reactions, people getting better at putting phone on mute in between so there is no audible feedback. Online requires a higher level listening set of skills and knowing how a panel works – if have not done a panel before may not be able to learn how one works.

Online is a lot of work/ a pain to see how people have issues on a phone that you would never have F2F.

PO6 - Q5. Critical

Process can introduce and reinforce skills that would make someone better in the future. For scientific review of proposals, there is no substitute for actually doing it. Begin small (dissertations, internal grants, etc), the skills learned are listening to someone make an argument and trying to understand strengths/weaknesses of their argument; Be able to read and distill proposal to key components – only learn by doing; If in busy panel (20+ panelists) that really helps/ important to see that general discussion of a proposal we cannot move forward – panelist can see they need to be more specific. Skills - unsure how would be taught – might be dry/ difficult to teach outside of panel review.

Prepares by reviewing journal articles. Really important to be able to read what others have written in their review. Not in classes, but in some classes where read each other’s work – not a lot of opportunities in formal education.

PO6 - Q6. Other/ Additional

A community is doing review process for an organization who wants to give money. Funding agency gives up some control over that … group of players involved – agency, program manager, panel lead, panelists, and proposer. All must be weighed when determining the funding awardees. Ex: [agency] had program to give resources to faculty at smaller institutions – these required extra thinking on part of panelists and funding agencies who took all this in.

**PO7 – 04/24/18**

PO7 - Q1. Experience

[Agency], ½ to 1/3 portfolio focus on grants to academic (short-term, nimble), other part of portfolio from labs (long term investment) use same peer reviewers; Experience building panels – big to small. Formats – used all formats imaginable, depends on nature of program/ proposal; single source proposals – large experiments in one program = long term renewal proposals.

In-person – bring review team in (leadership team), presents to review panel (more robust, get at how to do science at the labs); Boutique efforts (short term 2-4 year) study – very targeted – usually mail-in or virtual panel – can talk to each other/ conference line. This is prone to technological issues (drops, mute); Academic proposals usually a traditional review panel – we select, travel, go through each proposal one-by-one, write comments and use those to build a panel summary of each proposal.

There is a lot of variation – all use the same playbook – lawyers interpret differently.

[Agency] reach plus/minus degree of consensus; [Agency] – panel not reach consensus, [office] will make agency consensus

PO7 - Q2. Skills

Actually READ proposal – frustrating – get one sentence reviews not useful to make decisions or have the PI understand the issues; Assign primary reviewer until the end – primary writes the panel summary and presents the review – found people waited to find which proposals they really need to read; Diversity – gender, academic vs lab, field of science, racial, European vs American, career stage (early career are really close to the cutting edge vs big picture view held by later career scientists); As PO – get things out early (time commitment); Have panelists share scores – helps to normalize scores; Ask to talk about positives and negatives – hearing comments helps shape the score; Open-mindedness – willing to listen to others; Prepared

PO7 - Q3. F2F

Developed by participation – can be developed – review guidelines first before writing grant (spell check, layout, flow) – these things irritate reviewers; Experience teaches; No formal process that trains scientist on grantsmanship or reviewing proposals; Diversity – hearing different viewpoints, look at same problem with different lenses – good training experience; F2F training aspects is back and forth dialog, for multiple millions of dollars – long term proposals – without a narrative hard to understand what to expect, [agency] scientist used to this, think large scale, long term…devil is in the details – training here

Younger, newer scientist are good for diversity and teaches them how to be a successful grant seeker (PO7 sets a window of the number, not expected to be experts in all proposals they will get, look for COIs, bring in 1-2 experts in a proposal area and a generalist, tell them scores and comments should match – if score is a 9 there should not be 4 pages of negatives, PO7 tells them – write reviews and if you have questions can change and adjust scores at the panel “based on the discussion” and period of marinating; Some have questions about the review criteria – sometimes clarification is needed, some are concerned – what if my review is completely different from all others on the panel; First time reviewers will have bad proposal and feel guilty about the low scores. Need assurance that it is okay, there is such thing as a bad proposal.

Skills are the same no matter the format. Part of the enterprise of science is discussing, evaluating, and debating. With proposal review process – detail or quantity to write – get stuck on big things – what do you want to hear about your own science; Avoid sharp, pointed comments – avoid nit-picking and being overly negative; Live and breathe by the peer review process.

PO7 - Q4. Online

Development of skills – online – not a lot of contact – does homework for fit and confirms willingness. Just gets note saying review is done. Virtual – as a PM need to read body language – reviewer may go on and on because feel they need to say something. Hard to hear body language. Hard to know if someone need a little coaching, especially early career scientists. Video? – has never done video, would not advocate it because it adds another layer of complexity. Advantage to remote/ virtual – allows international reviewers, it doesn’t make sense to fly international reviewers in for a 1-day panel, for example.

Other cons with online – value-added thing for panelist is meet other scientists would never meet otherwise. Networking opportunities to collaborate on projects or get jobs. Virtual panels do not provide.

One of the most exciting parts of [PO7] job is bringing people together. Encourage panelists to conduct more networking at the location. Atypical for some PMs but reviewers and panelists appreciate, [PO7] sends thank you letter about one month out – it helps with CV and tenure/ promotion dossier – also makes them want to come back again.

PO7 - Q5. Critical

[no response]

PO7 - Q6. Other/ Additional

Only way science moves forward is by everyone contributing time to make sure review tasks are done. When panelists get into it they really get into it – issue is getting people to participate initially. Think for the most part people buy into process.

**PR1 – 04/4/18**

PR1 - Q1. Experience

35 years, served on 30-50 panels for research proposals; Also on review panels and chaired major activities such as (site visits); Variety of federal and non-US agencies, mostly [agencies]; In-person and online and blended

PR1 - Q2. Skills

Field dependent – in some cases clear need a broad knowledge rather than expertise in one area; Other panels much more focused – these require detailed expertise of current literature. Depends on the RFP and range of proposals gathered. Education proposals – impacts very different in structure from discipline-based RFPs; Time – invest time to understand the RFP or proposals tasked to look at. Effective reviewer requires time to think about what proposer is trying to do; Effective communicator – in panel discussion clear those who are better able to communicate have an advantage to make the case for/against a proposal. Definitely affects randomness of reviewers.

PR1 - Q3. F2F & Q4. Online (combined answer)

Easier in F2F to have side discussions – not allowed on many panel reviews except in the room; Sometimes a program officer will not allow side discussions, but they are essential in crafting a summary report; Different in virtual – no easy way to make it happen. Linear process, must force it. Not same as being able to talk about it.

Time and communication – time happens beforehand. Communicating virtually is just very different than F2F; regular… interviews – distance, in person different skill sets. Communication skills are different in each setting. Social skills – learn by watching and participating. Harder in online to learn, lack of clarity in virtual. If never served as a panelist learning to be effective does require invested time.

PR1 - Q5. Critical

Different skills needed. [some reviewers] cannot deal with the virtual stuff well – such as camera placement, sitting in front of the camera; Dealing with body language (cannot see it). At [PR1’s employer] – mentoring program – post-docs organize sessions “we would like this, this, and this”

Different kinds of panels – agencies assessing impacts – not discussed benefits to put on panels given time and investment. Different between agencies - [agency] – cannot say anything about serving except you did so, [agency] – much more open, accepted in [element of agency] is honor and info public on who servers.

When junior colleague – asks should I serve, [PR1] says yes – depends on their goals – going to submit to this agency then yes, if never going to submit proposal to the agency then rethink it. Skills learned from panel depends on the kind of panel – network not expanded from serving on virtual panel – for those trying to build network need to be in-person.

PR1 – Q6. Other/ Additional

When you have written enough proposals and see responses you learn what makes a good proposal. Role as a reviewer is to write points of proposal better than “elevator speech”. Best to ask “How do I give money…?” – shift from how do I get money/ where is it found.

**PR2 – 04/5/18**

PR2 - Q1. Experience

Served for multiple federal agencies F2F and online, or online one-off reviews and early stage- down to final stage (rank/ prioritize)

PR2 - Q2. Skills

Subject expertise – topic or how to do the research – depends on understanding research sufficiently; Use analytical tools – makes sense and how they are going about it – implementation; Scientific analysis – process/ research and implementation align; Analysis – careful reading, experience conducting, implements, or overseeing grants; Relative to participation on a panel – depends on the panel, if serve as a SME or expert in a component of the proposal; Play nice (academically disagree); Be concise; Well-prepared – understand the role of discussion lead or secondary (details); Impartial - honest about COI beforehand but separate personal vs professional

PR2 - Q3. F2F

Skills [PR2] gained – role of PO is essential in setting tone and providing instruction; Well prepared – lots of time stimulating, make you better grant work, good use of time; Comradery – should enjoy and take beyond reviews – connection about working together, network; Time well-spent

PR2 - Q4. Virtual

More difficult that F2F – harder to follow what people are talking about – attention; Difficult to do more than 4-5 hours online – fatigue (can do 2.5 days (8h/day) in F2F; No skills developed from doing online – problem online more difficult to disconnect from day-to-day activities; Review criteria closely – if you are leading, write good review and mark up proposal itself; Trust self – stick to opinion, have confidence but be open to change

PR2 - Q5. Critical

Setting dependent, prep same for both; Interpersonal and concentration skills; Examine budget – you are not making big budget decisions – implementation; Carrying out in a way that made sense – budget narrative + management of the grant; Management and fiscal skill

PR2 - Q6. Other/ Additional

[no response]

**PR3 – 04/5/18**

PR3 - Q1. Experience

Since 2002, two types of reviews for federal agencies and journal articles, also both F2F and online panels

PR3 - Q2. Skills

Most frustrating – know technical expertise but not reading reviews until last minute; Time management and prepared for time ahead; Technical expertise; Open-minded and flexible with looking at proposals outside comfort zone; Skills on review or participating – reach consensus and compromise with better exposure to that process, stubborn bad

PR3 - Q3. F2F and Q4. Online (discussed together)

Discuss and debate in F2F is easier than online or teleconference; Online didn’t help develop time management – more punctual, need to consider before participating; Subject matter expertise not developed in online

Another avenue to develop skills reviewing technical papers helps; Funding also look at PIs/ institution capable of conducting the research; Definitely learned fiscal stuff by being on a panel; Seed grant reviewer helps a bit, but federal level is a big learning curve. Online – convenience for [PR3], but more challenging to discuss/ debate online.

PR3 - Q5. Critical

Junior colleague advice – emphasize difference funding vs technical papers. 1^st^ review proposal looking at concrete evidence. In grant many not have the concrete evidence – knowing difference critical and something [PR3] learned as a panel reviewer.

PR3 - Q6. Other/ Additional

Good listener (academics love to talk) – need to be reminded of this; Know the difference between criteria for good grant v good technical paper; Knowing set-up for online (mic, lighting, camera position, connection)

**PR4 – 04/24/18**

PR4 - Q1. Experience

Reviewing for 20+ years; engaged in peer review of research proposals, grant proposals for [agencies], and have supported grant reviews for [countries] and a few other foreign research entities. Some panels have been F2F and some have been remote and others have involved the submission or written remarks plus teleconferences to discuss individual responses and come to a group consensus.

PR4 - Q2. Skills

Ability to quickly and correctly get sense of what is proposed and significance of the potential results, when there is interaction on a panel, there is variability among the reviewers on what is proposed to do and the significance of the contribution. Get right to heart of the matter; able to understand kind of contribution proposers are likely to make, challenging as may be reviewing a proposal that is not directly in the SME’s expertise.

Ability to make judgements on the team in the proposal and resources being requested are significant and appropriate to the task at hand; Think in an interdisciplinary way; Ability to express their view, must be written so chief reviewer can understand it and it is useful for the research team.

F2F or teleconference reviewer groups need to be able to debate strengths and weaknesses and come group decision. Must be a part of the discussion, not just sharing facts.

[Agency] panels can be split to share the load. Able to quickly write a useful and intelligent response. Light, useless review is not helpful and can discourage further submissions. Should provide enough feedback to authors of proposal to use as development opportunity. Ability to write a developmental review is important.

Interdisciplinary skills - encouragement/incentive to produce interdisciplinary proposals. Challenge - reviewers not trained nor inclined to adopt or appreciate interdisciplinary approaches. [Agency] encouraging interdisciplinary approaches, but challenged in putting together review panels that have the skills necessary to look at it from an interdisciplinary perspective. Consistent concern across all disciplines. Lots of frustration.

[Reviewers] must be accomplished in their field. Need to be solid scientists in their own right. Need to have written research proposals themselves so that they have been a part of the process. Best peer reviewers have lots of experience in working on grant proposals and being on peer review panels.

PR4 - Q3. F2F

Getting at the heart of the contribution, being able to hear people how they understand novelty and how they make judgments on how they see the novelty. The conversations and learning all the different approaches to making judgements on novelty. It can be nice to have a person who is very knowledgeable on the subject of the proposal but also being able to see the process on what …

Conversations and different perspectives on what it takes to do different types of projects. PR4 is a [type of] scientist with a particular kind of research tradition but an interdisciplinary field will have different research traditions so being able to understand all of those and why people ask for resources in different ways than my tradition. Being able to get quick insight on how “business” is being done in different traditions / models used in different disciplines. Can’t really have a class on that.

Process of creating interdisciplinary proposal allows for exposure for a variety of traditions. The best way to gain experience is by exposing people through the process of participating in reviews and creating proposal. The post doc positions in [physical] science vs social sciences.

Other value of being together is the developmental comments. [Agency] F2F peer review allows for a joint conversation among the panel that could be said to the proposer to make the proposals stronger. Allows for quick conversations to share comments with authors. F2F allows 5 to 10 minutes conversation on the proposal. Quickly, highly valuable feedback. May not be able to do so as effectively if not F2F.

PR4 - Q4. Online

Read materials, fill out the form, send in comments. That has some limited value. Allows [PR4] to see different models and different ways to review, less value to the reviewer, the reviewer entity. There has been no engagement between the reviewers about the work.

1st model - It’s a community service. I want to help develop research programs.

2nd model - The aggregation of the comments is done by the moderator.

3rd model – No engagement with other reviewers due to volume and timeline – less satisfying for the reviewer and may be less useful?

Harder to hide when you’re F2F. Hide = hasn’t actually read the proposal – not a skill, but a principle. It takes time. If you committed to the job, do the job and show up. Tends to happen less in F2F.

Really important to consume what a proposal is putting forward and claiming. Comprehension is very important. There isn’t a lot of time. It’s even more important in virtual meetings as there isn’t as much time. 1-2 hours on the phone is exhausting, but in F2F, sometimes get a few minutes to relook at things.

The better the proposal the easier the job of the reviewer.

Be able to get to a conclusion and be able to place the proposals in one of three piles: Contenders, possible contenders, and no thank you. Yes and maybes is where most of the work has to happen. Quickly coming to a conclusion on what should be on the bubble then focus on the details of the contenders.

PR4 - Q5. Critical skills

Group model building / facilitation – one frustration is that they like what they are doing but I am not a person who can come up with answers quickly. I need time to think about what I am reading or what I want to say. I am thoughtful and careful and specific and would be happy to give comments in a day. People who may cannot respond quickly or need time to think may not be naturally attracted to the review process.

Challenge is that the person who knows the most about what I am trying to model is not a person who is in a group modeling exercise at the pace of the group activity. This isn’t a skill but as a particular characteristic of people. How do we get to the benefit of people like that? What are we missing if we don’t include these people?

In virtual review, need to have a tight plan to move people through and capture results. The most important part of virtual with teleconference is planning the teleconference. How do we capture results? Must be very concrete and have timelines.

A 2 day review panel – the planning of the days is very important. It’s environmental – break time, that the systems work, must have a clear map of what is going to happen and how much time is spent on the work. Do not push all to the end. The management of the panel is as important as a virtual teleconference.

Logistics, environment, time management

PR4 - Q6. Other/ Additional

The other important trait is that people recognize the value of the comments of their peers. They see themselves as part of a group process. They don’t have to win every point, they take it seriously, (find best proposals to fund and providing developmental comments, must work well with others to 1) find best science and 2) helping develop future scientists.

Sometimes the best scientists aren’t the best reviewers. [PR4] sits on [agency] committee that is talking about the problems that the large facilities grants have been having over the past few years. For example, a few [dollar amount] to build a [type of] facility. Unfortunately, a number of the facilities are having significant problems. The reason is that the idea was good, the science was important, but they didn’t pay attention to the management model; very little attention or priority given on if it could be built and managed. From a reviewing perspective, the proposers must be able to deliver on the idea. The ability to make judgement on the science as well as the project management. [Agency] is looking at new requirements for proposals [to ensure there is a management plan]. It’s the science plus the capability to deliver.

[Doctoral] program [PR4] is a part of has a boot camp on how to write a conference proposal and serve as reviewers of conference proposals and allows individuals to gain experience.

**PR5 – 04/26/18**

PR5 - Q1. Experience

Reviewer external to panels for a long time, for various agencies (international too), [agencies. Also on panels for [agency] where met in [location] together or virtual.

PR5 - Q2. Skills

Process of receive grants, why process of PR/ goals understand role as reviewer, know general area, in panel – writing yourself and sometimes evaluating other reviews.

How learned – typically sent instructions for the review, or a guided form (more recently). Some programs changed things ([agency]-new-form very specific questions used to be a page or two on factors to consider – now targeted questions). [Agency] has distinct categories in which they request information and typically provide guidelines. Understand PR and role – different in panel setting – yes, they remind everyone of process ([agency] shows PPT first). Serving on panel and listening to others get clues and system – cultural aspect to serving on a panel. Other skills critical – review proposal first to ensure you have general knowledge to effectively review it. Decision – expertise in some part of the proposal to judge quality.

PMs always are looking for reviewers. Organizations tend to pick people from them or senior scientists. Start soon – lots to do, read carefully and think of guidelines. Do not announce you are on [agency] panel – should be quiet – this avoids bias and identifiers. Would give advice on how panels have been run in [PR5s] experience. Tell you will be asked to review and engage in those but others not “officially” part of team reviewing, okay to chime in. If person is shy would encourage them to speak up. Skills of PR process – have to read/write English proficiently, be positive and objective, constructive criticism, back up argument in support of proposal.

PR5 - Q3. F2F & Q4. Online

Online – set up to mute mic – unmute when speak. Occurred that shy person will be called upon by moderator and bold people need to remember to allow others to talk. Social cohesion in F2F.

Skills are similar ([PR5] has less experience with online). PMs say learn about writing proposal, meet names in the field. F2F is tougher on single parent or other home obligations. Observations from other [people] and when [PR5s] children were young – one-sided conversation.

PR5 - Q5. Critical

[no response]

PR5 - Q6. Other/ Additional

[no response]
