# Supplementary material for: What makes an effective grants peer reviewer? An exploratory study of the necessary skills
Source: PLoS One. 2020 May 13;15(5):e0232327. doi: 10.1371/journal.pone.0232327 (PMC7219739; doi:10.1371/journal.pone.0232327)
Supplement: S3 Appendix — (PDF) [file pone.0232327.s003.pdf]

Peer Review Skills Development in Multiple Panel Review Formats

Informed Consent

Title of research study: Peer Review Skills Development in Multiple Panel Review Formats
Investigators: Tiffani R. Conner (PI), Miriam L.E. Steiner Davis (Co-PI), Leslie Shapard (Sponsor)

Purpose
Peer reviewers are experts who review and evaluate research. Peer reviewers sometimes review research submitted for publication or funding independently. In other cases, to assist an agency in deciding which research to support, a group of peer reviewers (a review panel) discuss together their review of proposed research in the form of research proposals or grant proposals. These people are review panelists.

We are interested in **your experience as a review panelist**. In particular, we are interested in the **competencies** you think are necessary to be an effective review panelist and **how you think they are best developed**. This survey should take 10 minutes to complete.

Risks and Benefits: This research involves minimal risk. Benefits for the scientific community include further understanding of peer review skills and how they are obtained. However, there are no direct benefits to you personally from taking part in this research.

Participation: Participation is voluntary. You may choose to quit at any time without any negative consequences.

Anonymous or Confidential: All survey data is anonymous.

Who will see my information? In research reports, there will be no information that will make it possible to identify you. Research records will be stored securely and only approved researchers and the Oak Ridge Sitewide Institution Review Board will have access to the records.

Who to contact:
If you have questions, concerns, or complaints, you can talk to the research team at:

Dr. Tiffani R. Conner
ORISE
(865) 415-8049
E-mail: Tiffani.Conner@orau.org

This research has been reviewed and approved by an Institutional Review Board (the Oak Ridge Sitewide Institutional Review Board or "ORSIRB"). You may talk to them at (865) 574-4359 or ORSIRB@orau.org if:

1. Your questions, concerns, or complaints are not being answered by the research team.
2. You cannot reach the research team.
3. You want to talk to someone besides the research team.
4. You have questions about your rights as a research subject.
5. You want to get information or provide input about this research.
6. Or for any reason you wish to talk to the ORSIRB.

By clicking 'Next' below you are confirming and accepting the Informed Consent and agreeing to participate in this research.

Peer Review Skills Development in Multiple Panel Review Formats

For each question, please choose the response choice that best reflects your experience or opinion.

1. Is the United States your country of residence?

- Yes
- No

2. Have you served as a review panelist for research proposals or grant proposal decisions?

- Yes
- No

Peer Review Skills Development in Multiple Panel Review Formats

3. Based on your experience, please indicate your level of agreement that the following competencies are important to being an effective review panelist.

	Strongly disagree	Disagree	Neutral	Agree	Strongly agree
Subject matter expertise in an area(s) related to the panel.	○	○	○	○	○
Familiarity with panel reviews.	○	○	○	○	○
Broad scientific understanding is important to being an effective review panelist.	○	○	○	○	○
Familiarity with the specific agency's peer review process is important to being an effective review panelist.	○	○	○	○	○
Preparedness (e.g. have read the solicitation, the directions, and any proposals assigned) is important to being an effective review panelist.	○	○	○	○	○
Impartiality is important to being an effective review panelist.	○	○	○	○	○
Analytical thinking is important to being an effective review panelist.	○	○	○	○	○
Open to others' opinions or ideas is important to being an effective review panelist.	○	○	○	○	○
Clear and concise writing is important to being an effective review panelist.	○	○	○	○	○
Active and attentive listening is important to being an effective review panelist.	○	○	○	○	○
Open to novel research ideas is important to being an effective review panelist.	○	○	○	○	○
Sensitivity towards bias(es) is important to being an effective review panelist.	○	○	○	○	○
Confidence in one's own opinion or ideas is important to being an effective review panelist.	○	○	○	○	○

Peer Review Skills Development in Multiple Panel Review Formats

4. Based on your experience, please indicate your level of agreement that the following competencies are important to being an effective review panelist. The ability to:

	Strongly disagree	Disagree	Neutral	Agree	Strongly agree
...put proposed research in context.	○	○	○	○	○
...articulate ideas clearly.	○	○	○	○	○
...sustain attention to the task.	○	○	○	○	○
...interpret body language including facial expressions.	○	○	○	○	○
...build rapport with other panelists.	○	○	○	○	○
...redirect conversations politely.	○	○	○	○	○
...stay on topic.	○	○	○	○	○
...collegially voice dissent or disagreement.	○	○	○	○	○

5. Please provide any additional comments about the competencies necessary for an effective review panelist.

Peer Review Skills Development in Multiple Panel Review Formats

Review panels occur in different settings. We are interested in two specific settings: Face-to-Face (or "in-person") and Virtual (online, synchronous).

In Face-to-Face (F2F) panel reviews, ALL review panelists meet in person at the same time and in the same space.

In Virtual panel reviews ALL review panelists participate at the same time but NOT ALL IN THE SAME PLACE (some may be together in the same place, some may be participating online via conference call, or all may be participating online at the same time).

6. Please indicate which review panel setting you believe best helps develop or improve each of these competencies. Improved:

	More by virtual participation	Equally by virtual or Face-to-Face participation	More by Face-to-Face participation	Not improved by either format
...subject matter expertise in an area(s) related to the panel.	○	○	○	○
...familiarity with panel reviews.	○	○	○	○
...broad scientific understanding.	○	○	○	○
...familiarity with the specific agency's peer review process.	○	○	○	○
...preparedness (e.g. have read the solicitation, the directions, and any proposals assigned).	○	○	○	○
...impartiality.	○	○	○	○
...analytical thinking.	○	○	○	○
...open to others' opinions or ideas.	○	○	○	○
...clear and concise writing.	○	○	○	○
...active and attentive listening.	○	○	○	○
...open to novel research ideas.	○	○	○	○
...sensitivity towards bias(es).	○	○	○	○
...confidence in one's own opinion or ideas.	○	○	○	○

Peer Review Skills Development in Multiple Panel Review Formats

Review panels occur in different settings. We are interested in two specific settings: Face-to-Face (or "in-person") and Virtual (online, synchronous).

In Face-to-Face (F2F) panel reviews, ALL review panelists meet in person at the same time and in the same space.

In Virtual panel reviews ALL review panelists participate at the same time but NOT ALL IN THE SAME PLACE (some may be together in the same place, some may be participating online via conference call, or all may be participating online at the same time).

7. Please indicate which review panel setting you believe best helps develop or improve each of these competencies. Improved the ability to:

	More by virtual participation	Equally by virtual or Face-to-Face participation	More by Face-to-Face participation	Not improved by either format
...put proposed research in context.	○	○	○	○
...articulate ideas clearly.	○	○	○	○
...sustain attention to the task.	○	○	○	○
...interpret body language including facial expressions.	○	○	○	○
...build rapport with other panelists.	○	○	○	○
...redirect conversations politely.	○	○	○	○
...stay on topic.	○	○	○	○
...collegially voice dissent or disagreement.	○	○	○	○

Peer Review Skills Development in Multiple Panel Review Formats

8. Based on your experience, please indicate how much each experience below has improved your review panelist competencies.

	Did not improve	Minimally improved	Somewhat improved	Strongly improved	I have not experienced this
Observation of other panelists	○	○	○	○	○
Listening to panelists make arguments	○	○	○	○	○
Sharing my thoughts during discussions	○	○	○	○	○
Being the chair / responsible for running a discussion	○	○	○	○	○
Casual discussions with senior colleagues	○	○	○	○	○
Reading reviews of my own research proposals	○	○	○	○	○
Being mentored by colleagues experienced in panel reviews	○	○	○	○	○
Mentoring others concerning participation in panel reviews	○	○	○	○	○
Serving as a peer reviewer of manuscripts for publication (unrelated to panel review participation)	○	○	○	○	○
Participating on more than one panel	○	○	○	○	○
Writing / submitting research proposals myself (e.g. to other funding opportunities)	○	○	○	○	○
Academic training (e.g. graduate programs, workshops)	○	○	○	○	○
Training / instructions from funding agencies	○	○	○	○	○

9. What other experiences help develop or improve review panelist competencies?

Peer Review Skills Development in Multiple Panel Review Formats

We are interested in the review panel formats in which you have participated.

In Face-to-Face panel reviews, ALL review panelists meet in person at the same time and in the same space.

In Virtual panel reviews, ALL review panelists participate at the same time but NOT ALL IN THE SAME SPACE (some may be together in the same place, some may be participating online, or all may be participating online at the same time).

10. How many FACE-TO-FACE panel reviews have you participated in?

- 0
- 1 - 5
- 6 - 15
- 16 - 25
- 26 - 50
- More than 50

11. How many VIRTUAL panel reviews have you participated in?

- 0
- 1 - 5
- 6 - 15
- 16 - 25
- 26 - 50
- More than 50

Peer Review Skills Development in Multiple Panel Review Formats

12. Please indicate your type of employment.

- ☐ Government agency/office
- ☐ National lab
- ☐ University
- ☐ Corporation
- ☐ Non-profit company
- ☐ Other

13. Which educational degree(s) have you earned?

Select all that apply.

- ☐ Associate's
- ☐ Bachelor's
- ☐ Master's
- ☐ Doctorate
- ☐ Professional (e.g., JD, MD, DDS, DVM)
- ☐ Other

14. Please indicate your career stage.

- ○ Early (1-10 years)
- ○ Middle (11-20 years)
- ○ Senior (21+ years)

Peer Review Skills Development in Multiple Panel Review Formats

15. What is your field of work?

- Biology
- Chemistry
- Computer Science
- Engineering
- Environmental Sciences
- Materials Science
- Mathematics
- Physics
- Other

16. Please indicate your sex.

- Male
- Female
- Prefer not to answer
- Other

17. What is your age range?

- 18 - 24
- 25 - 34
- 35 - 44
- 44 - 54
- 55 - 64
- 65+
- Prefer not to answer

Peer Review Skills Development in Multiple Panel Review Formats

18. For which agencies have you participated in panel reviews of research/ grant proposals?

Select all that apply.

- ☐ AIBS (American Institute of Biological Sciences)
- ☐ Bill & Melinda Gates Foundation
- ☐ CDC (Centers for Disease Control)
- ☐ DHS (Department of Homeland Security)
- ☐ DOD (Department of Defense)
- ☐ DOE (Department of Energy)
- ☐ DOI (Department of Interior)
- ☐ DOJ (Department of Justice)
- ☐ EPA (Environmental Protection Agency)
- ☐ International Agency(ies)
- ☐ NASA (National Aeronautics and Space Administration)
- ☐ NIH (National Institutes of Health)
- ☐ NIST (National Institute of Standards and Technology)
- ☐ NOAA (National Oceanic and Atmospheric Administration)
- ☐ NSF (National Science Foundation)
- ☐ Robert Woods Johnson Foundation
- ☐ USDA (United States Department of Agriculture)
- ☐ William T. Grant Foundation
- ☐ Other

Peer Review Skills Development in Multiple Panel Review Formats

Thank you for your interest in our research.

Please exit the survey by clicking the "x" normally located in the top right corner of your web browser.

Peer Review Skills Development in Multiple Panel Review Formats

Thank you for taking the time to participate in this research. Please click 'Submit'.
